# Intra-host variation and transmission dynamics of SARS-CoV-2 Omicron outbreaks in Shandong, China

**DOI:** 10.1128/msphere.00355-25

**Published:** 2025-09-22

**Authors:** Xuemin Wei, Qi Gao, Yuhao Wang, Xinyi Gao, Zengqiang Kou, Xiujun Li, Yifei Xu

**Affiliations:** 1 Department of Microbiology, School of Public Health, Cheeloo College of Medicine, Shandong University 66555, Jinan, Shandong, China; 2 Shandong Center for Disease Control and Prevention 373247, Jinan, Shandong, China; 3 Shandong Provincial Key Laboratory of Intelligent Monitoring, Early Warning, Prevention and Control for Infectious Diseases, Jinan, Shandong, China; 4 Suzhou Research Institute of Shandong University 731745, Suzhou, Jiangsu, China; Duke-NUS Medical School, Singapore, Singapore

**Keywords:** intra-host diversity, iSNV, SARS-CoV-2, co-mutation pattern, evolution

## Abstract

Investigating the intra-host diversity of severe acute respiratory syndrome coronavirus 2 (SARS-CoV-2) is essential for understanding its transmission and the emergence of new variants. However, there is limited insight into SARS-CoV-2 intra-host diversity and the extent to which shared intra-host single nucleotide variants (iSNVs) occur among samples without epidemiological links. To characterize intra-host diversity, we analyzed sequencing data from 803 samples across four Omicron transmission clusters. The potential co-mutation patterns formed by shared iSNVs contributed to regions in the genome with elevated iSNV density. Most samples did not share iSNVs. Even among the sample pairs that did share at least one iSNV, 24.4% originated from different transmission clusters. For shared iSNV sites that can become fixed as single nucleotide polymorphisms (SNPs), iSNVs cluster within the phylogenetic tree, with branches supporting the same variants as SNPs. This observation suggests that iSNVs likely serve as reservoirs for SNPs. Additionally, the BA.1.1 samples carried iSNVs identical to the characteristic mutations of BA.2 and BA.2.3. These findings provide important insights into the evolution and transmission inference of SARS-CoV-2.

**IMPORTANCE:**

Understanding the mechanisms behind viral evolution and transmission is crucial, as novel SARS-CoV-2 variants continue to emerge and spread worldwide. Viral evolution is driven not only by variants that circulate globally but also by mutations arising within individual hosts, resulting in the emergence of iSNVs. The role of iSNVs in shaping SARS-CoV-2 evolution and transmission remains poorly characterized. Our results showed a significant enrichment of shared iSNVs in high-density genomic regions, potentially contributing to the formation of co-mutation patterns. However, the presence of shared iSNVs in samples lacking epidemiological links indicates that they alone are insufficient for accurately reconstructing transmission routes. Instead, iSNVs may act as a reservoir for the emergence of single nucleotide polymorphisms. Our study offers new insights into the evolution of SARS-CoV-2 and the interpretation of transmission from sequencing data.

## INTRODUCTION

Since its emergence in November 2019, severe acute respiratory syndrome coronavirus 2 (SARS-CoV-2) has continuously evolved through mutations and recombination events, leading to the emergence of multiple variants of concern such as Alpha, Delta, and Omicron (1–3). These variants are characterized by distinct mutations that enhance transmissibility, immune evasion, or pathogenicity, resulting in successive waves of COVID-19 infections worldwide (1, 4). The continuous emergence and global spread of such variants highlight the critical need to understand the mechanisms driving viral evolution and transmission.

The evolution of the virus occurs not only through globally circulating variants but also through mutations within individual hosts, leading to the emergence of intra-host single nucleotide variants (iSNVs) (4). Compared to single nucleotide polymorphisms (SNPs), iSNVs encompass a broader spectrum of genetic mutations, offering deeper insights into viral diversity and evolutionary dynamics. For example, the D614G of SARS-CoV-2, which improved viral replication and transmission, was initially detected as an iSNV in samples before becoming recognized as an SNP (5–9). While intra-host diversity of SARS-CoV-2 has been observed, research on this aspect remains limited (10).

Analyzing transmission patterns during outbreaks is crucial for understanding pathogen spread and designing targeted public health measures. Combining phylogenetic analysis with epidemiological investigations allows for the identification of the origins and transmission clusters of pathogens (11–13). However, consensus sequences, which reflect only the dominant viral lineage within a host, offer limited resolution for transmission analysis. In contrast, the analysis of intra-host diversity may provide a more precise approach to studying viral transmission (14, 15). Despite its potential significance, the characteristics of SARS-CoV-2 intra-host diversity and the extent to which shared iSNVs occur among samples without epidemiological links remain insufficiently explored (16, 17).

Here, we analyzed sequencing data from 803 samples across four transmission clusters that occurred during the Omicron epidemic in Shandong Province in March 2022. We aimed to characterize the features of intra-host diversity, investigate the likelihood of shared iSNVs arising among samples without epidemiological links, and explore the transition of iSNVs into SNPs. Our findings enhance our understanding of SARS-CoV-2 variation in individual hosts and highlight key considerations for inferring transmission using shared iSNVs.

## MATERIALS AND METHODS

### Data collection

The sequencing data of 803 SARS-CoV-2 Omicron samples, collected from public health laboratories in Shandong as part of routine surveillance, were obtained from our previous report (18). These samples were obtained from four transmission clusters, SD-1–SD-4, during the Omicron epidemic in Shandong between 1 March and 27 March 2022. These four local transmission clusters were identified by a previous study based on genome phylogeny and public health investigation results. These four clusters belonged to three genetic sublineages of the Omicron variant: BA.1.1 (SD-1), BA.2 (SD-2), and BA.2.3 (SD-3 and SD-4).

### Genome sequencing

All samples were collected by personnel certified through unified biosafety training and operational protocols. Viral RNA extraction, cDNA synthesis, and sequencing were performed as previously described (18). Viral RNA was extracted using the QIAamp Viral RNA Mini Kit (Qiagen, Hilden, Germany). The extracted RNA was reverse transcribed and amplified using the ULSEN 2019-nCoV Whole-Genome Capture Kit (MicroFuture Technology, Beijing, China). The resulting cDNA was purified using AMPure XP beads (Beckman Coulter, USA) at a 1:1 ratio and quantified with the Qubit 1X dsDNA HS Assay Kit (Thermo Fisher Scientific, USA), following the manufacturer’s instructions. Libraries were prepared from 0.2 ng/µL cDNA using the Nextera XT Library Prep Kit with Nextera XT Indexes (Illumina, San Diego, CA, USA). Sequencing was performed on Illumina NextSeq 2000 or MiSeq platforms.

### Calling of iSNVs

Sequencing reads were mapped to the reference genome (Wuhan-Hu-1, NCBI NC_045512.2) using BWA-MEM version 0.7.17 (19). Only properly paired reads were retained for the downstream analysis. PCR-duplicated reads were removed using Picard MarkDuplicates (version 2.10.10) (http://broadinstitute.github.io/picard). Base composition of each position was summarized from the mapping profile using pysamstats (version 1.1.2, https://github.com/alimanfoo/pysamstats). To enable accurate iSNV detection while minimizing false positives from sequencing or alignment errors, the following filtering criteria were applied, consistent with established practices: (i) bases with a Phred-scaled base quality score <20 were excluded to improve the specificity of low-frequency variant calling (20, 21). (ii) Sites required a minimum depth ≥100× to ensure sufficient power for detecting variants (22). (iii) The variants required ≥10 supporting reads to distinguish them from sequencing noise (22). (iv) iSNVs with strand bias greater than 10-fold were removed to avoid strand-specific artifacts (20). (v) Only variants with allele frequencies (AFs) between 3% and <70% were retained, ensuring that low-frequency variants (AF ≥ 3%) exceed typical sequencing error rates (17, 22–24). (vi) Genomic regions containing ≥5 iSNVs within a 50 bp window were excluded to minimize false positives from local misalignment (25). To avoid recurring sequencing errors, iSNVs with consistently low AF identified in multiple samples were excluded (Fig. S1) (23). SNPs were defined as variants meeting the following criteria: (i) depth of coverage ≥ 10; (ii) AF ≥ 70%. Using these rigorous, literature-supported criteria, iSNVs and SNPs were identified from our sample set. To rule out cross-sample contamination, we specifically compared the genomic positions of identified iSNVs with SNP sites from our samples. Contamination typically results in spurious iSNVs overlapping SNP positions due to cross-sample mixing. In our data set, only 8.37% (123 iSNVs) overlapped with SNP positions—a much lower proportion than previously reported (26)—indicating that contamination did not appreciably affect our iSNV calls. The iSNV density in a given region was calculated by dividing the total number of identified iSNVs by the number of samples and then further dividing by the length of the region.

The consensus sequences were derived from prior research (18). These sequences were generated with a minimum of 10-fold mapping coverage and supported by at least 70% of reads at each position. Consensus sequences with more than 90% genome coverage were used for downstream analysis. The genetic clade for each SARS-CoV-2 sequence was determined using Nextclade version 1.11.0 (https://clades.nextstrain.org).

### Evaluating iSNV genomic distance distributions

iSNV positions were simulated in R using the runif function, which utilizes a uniform distribution based on the number of iSNVs. Both actual and simulated iSNVs were ranked, and genomic distances between neighboring mutant pairs were calculated. It was found that the distances between simulated iSNVs followed a Poisson distribution. To evaluate the distribution of distances between adjacent iSNVs, the Kolmogorov-Smirnov test was used to compare the observed distribution with the expected distribution under a Poisson model.

### Identification of co-infection cases

Co-infection cases must satisfy three criteria: (i) a sample must contain featured mutations from more than one sublineage; (ii) the AF of mutations within the same sublineage should be similar; (iii) the total AF across all detected sublineages should be close to 100% (27). In March 2023, 11 BA sublineages (BA.1, BA.1.1, BA.1.14, BA.1.15, BA.1.1.1, BA.1.1.2, BA.2, BA.2.2, BA.2.3, BA.2.9, and BA.2.10) were circulating in Shandong Province. Their featured mutations were collected (https://outbreak.info/situation-reports), and the AF of polymorphic sites was analyzed for each sample.

### Analysis of potential co-mutation patterns

A network of the shared iSNVs was constructed using Cytoscape version 3.9.1 (28). An edge-weighted, spring-embedded model was used to determine the layout. In the network, each node represented a sample, and two nodes were connected through an edge if samples shared ≥1 iSNVs. Samples that shared more iSNVs were closer in network distance. There were 229 nodes and 994 edges.

Potential co-mutation patterns were further evaluated using the Pearson correlation coefficient (PCC) and the significance level (*P*-value) obtained from linear regression analysis. The coefficients of linear correlation between two shared iSNVs were evaluated by PCC, with a value between −1.0 and 1.0. There was a strong correlation between the two iSNVs when the absolute value of PCC was between 0.7 and 1. The relationship between one or more independent and dependent variables was modeled by linear regression.

### Phylogenetic association of iSNVs and SNPs

If an iSNV was detected at a specific genomic site in a sample, and a corresponding SNP (with the same base pair substitution, not just the same genomic location) was found at the same site in other samples, these sites were designated potential fixed sites. To prevent misclassification of lineage-defining mutations (relative to the Wuhan-Hu-1) as SNPs in subsequent analyses, all genomic positions corresponding to the featured mutations defining the Omicron sublineages observed in our study (BA.1.1, BA.2, and BA.2.3) were systematically excluded. These featured mutations were predominantly curated from Outbreak.info (https://outbreak.info/situation-reports). The consensus sequences obtained from GISAID (https://gisaid.org/) and those from the Omicron outbreak in Shandong, China, were aligned using MAFFT version 7.310 (29). A maximum likelihood phylogenetic tree was constructed based on SARS-CoV-2 coding region sequences using IQ-TREE version 2.0.3 with the best-fit substitution model determined by the software (30). Branch support was assessed with ultrafast bootstrapping. The final tree was rooted using the reference sequence, after which the reference sequence, all GISAID isolates, and sequences not included in this study were pruned. When an iSNV corresponded to an SNP (defined by the base pair involved, not just the site), ancestral state reconstruction was performed on the consensus trees using ClonalFrameML to identify all branches on which that substitution had occurred (31). The patristic distance from each tip in the tree to the midpoint of the closest one of these branches was calculated. A one-tailed Mann-Whitney *U* test was then used to assess the association between the presence of iSNV in a sample and this distance. Multiple testing was controlled for each using the Benjamini-Hochberg adjustment. These analyses were done both on an individual site level and across all sites of interest.

## RESULTS

### Distribution of iSNVs among samples

**Fig 1:**
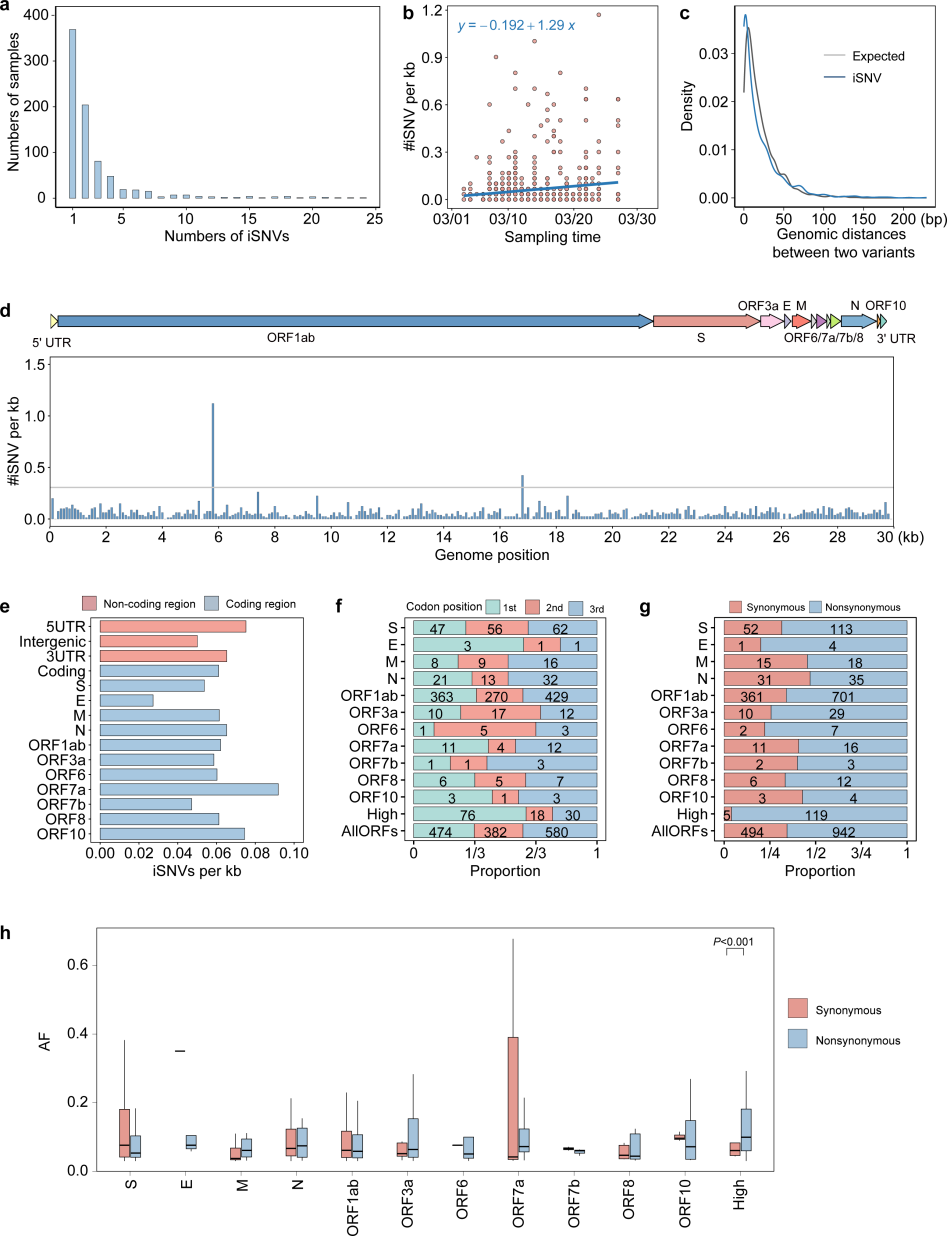
Distribution of iSNVs in SARS-CoV-2 Omicron variant. (**a**) The number of identified iSNVs per sample. (**b**) Distribution of the number of iSNVs/kb against the date of sample collection. (**c**) Distribution of genomic distance between two variants in observed and expected iSNVs. The blue line represents the observed iSNVs, while the gray line shows the expected distribution modeled by a Poisson process. (**d**) Distribution of the number of iSNVs/kb in the genome, counted using a window of 100 bp. The gray line represents five times the mean iSNV density in the genome. (**e**) Distribution of the number of iSNVs/kb in coding and non-coding regions. (**f**) Distribution of iSNVs at codon positions. High represents high-density regions. The number of iSNVs in each category is marked on the corresponding bar. (**g**) Proportion of nonsynonymous and synonymous iSNVs for each gene, high-density regions, and the combined ORFs (AllORFs). (**h**) Box plots of AF for non-synonymous and synonymous iSNVs.

We retrieved sequencing data for 803 SARS-CoV-2 Omicron samples from the Shandong epidemic between 3 March and 27 March 2022. The number of iSNVs in each sample was not affected by sequencing depth or Ct values (*R*-square [*R*^2^] = 0.14 for sequencing depth; *R*^2^ = 0.10 for Ct values of ORF1ab gene; *R*^2^ = 0.08 for Ct values of N gene; Fig. S1a through c), suggesting that the identified iSNVs were unbiased to sequencing data. We identified 1,470 iSNVs at 1,133 sites. The median number of iSNVs per sample was one (Fig. 1a). About 54.05% of samples (434 of 803) harbored at least one iSNV in comparison with the reference genome. We found a steady increase in the density of iSNVs over time during the epidemic, increasing from 0.03 to 0.23 iSNVs/kb within 24 days (Fig. 1b).

### Distribution of iSNVs across the genome

We investigated the distribution of iSNVs across the SARS-CoV-2 genome and found an overall iSNV density of 0.06 iSNVs/kb (Fig. 1d). The iSNV density of the 5′ UTR, 3′ UTR, and intergenic region was 0.08, 0.07, and 0.05 iSNVs/kb, respectively (Fig. 1d). We identified 1,436 (97.69%) iSNVs in the coding regions, which cover 97.85% of the genome. Most iSNVs (1,062, 73.96%) were present in the ORF 1ab, followed by the S gene (165 iSNVs, 11.49%) and N gene (66 iSNVs, 4.60%). After normalization by gene length, the ORF7a gene showed the highest iSNV density at 0.09 iSNVs/kb (Fig. 1e).

To determine if selection pressure influenced the distribution of iSNVs across the genome, we calculated the genomic distances between allele pairs. The fitted density curve showed a significant difference from randomly generated mutations (Kolmogorov-Smirnov test, *P* < 0.001; Fig. 1c), indicating a non-stochastic distribution of iSNVs. We also analyzed iSNV distribution across codon positions for all genes. The third codon position of ORF1ab had the highest iSNVs, followed by the first, with the second having the lowest (Fisher’s exact test, *P* < 0.05; Fig. 1f). The N gene showed more iSNVs at the third codon position than at the second (Fisher’s exact test, *P* < 0.05) and exhibited a lower nonsynonymous/synonymous ratio compared to other regions of the viral genome (1.13 vs 1.91, Fisher’s exact test, *P* = 0.03; Fig. 1g). This suggested that the N gene may be subject to stronger negative selection than other genomic regions.

Interestingly, we identified two distinct regions (referred to as high-density regions) in the ORF1ab gene at positions 5,700–5,800 and 16,700–16,800. The iSNV density in these two regions (1.12 and 0.42 iSNVs/kb) was 19 and 7 times higher than the overall iSNV density in the whole genome. In these high-density regions, the number of iSNVs at the first codon position was higher than at the second and third codon positions (Fisher’s exact test, *P* < 0.05; Fig. 1d). Moreover, the non-synonymous/synonymous iSNV ratio in these regions (23.8) was significantly higher than that of other regions of the viral genome (1.7, Fisher’s exact test, *P* < 0.001). While we did not observe a significant difference in AF between non-synonymous and synonymous iSNVs (Mann-Whitney *U*-tests, *P* = 0.367), the median AF of non-synonymous iSNVs (0.10) was higher than that of synonymous iSNVs (0.06, Fig. 1h). These results suggested that the iSNVs in high-density regions might be under stronger selection pressure.

### Potential co-mutation patterns in high-density regions

**Fig 2:**
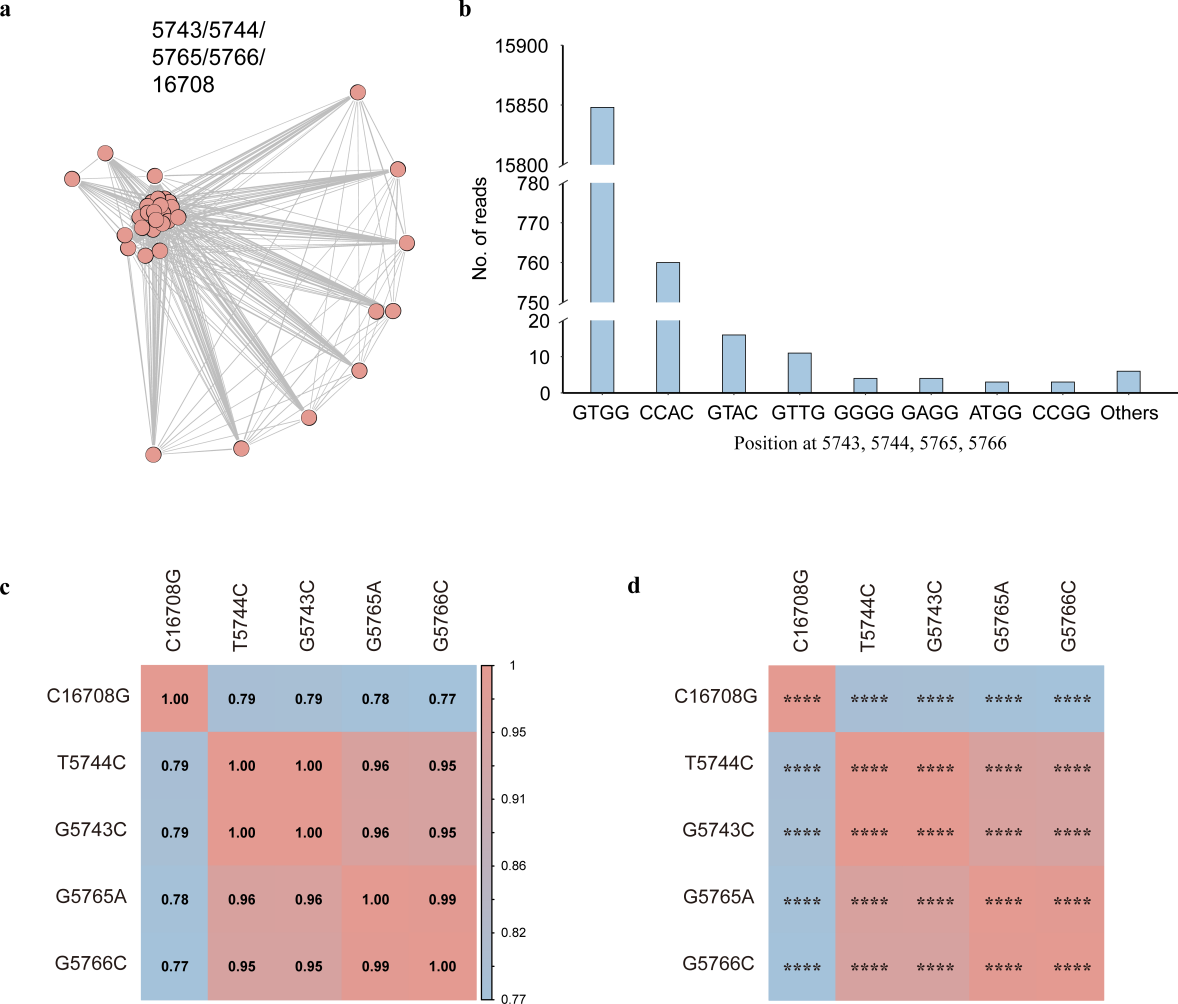
A potential co-mutation pattern consisted of the five non-synonymous shared iSNV sites (G5743C, T5744C, G5765A, G5766C, and C16708G). (**a**) Network analysis of the five non-synonymous shared iSNV sites. Nodes denoted samples, and two nodes were connected through an edge if samples shared ≥1 iSNVs. The shared iSNVs that caused the cluster were shown on the top of the network. (**b**) Reads distribution of 5,743, 5,744, 5,765, and 5,766 positions. (**c**) The correlation heatmap of the five non-synonymous shared iSNV sites. (**d**) Regression analysis of the five non-synonymous shared iSNV sites showed that the cluster denoted the statistical significance: ****P < 2.5 × 10^−87^.

We analyzed the number of iSNV sites and the proportion of shared iSNVs within high-density regions. The number of iSNV sites in high-density regions (0.06 iSNV sites/kb) was similar to that in other regions of the viral genome (0.05 iSNV sites/kb). Notably, the proportion of shared iSNVs (96.8%) among all iSNVs in high-density regions was significantly higher compared to other regions of the viral genome (22.9%; Fisher’s exact test, *P* < 0.001). The majority of these shared iSNVs within the high-density regions formed potential co-mutation patterns, as revealed by the shared iSNV network (Fig. 2a; Fig. S3), with shared nonsynonymous iSNVs—G5743C, T5744C, G5765A, G5766C, and C16708G—forming a significant cluster.

We then characterized co-occurring mutations at four genomic positions (5,743, 5,744, 5,765, and 5,766) across 752 high-coverage SARS-CoV-2 samples (≥100× at all target sites). After excluding reads with no mutations at these sites (the GTGG haplotype), the most common pattern observed was CCAC, corresponding to simultaneous mutations G5743C, T5744C, G5765A, and G5766C. This indicated that these shared iSNVs appeared together in the same virus strain (Fig. 2b). The reads exhibiting the CCAC pattern at four sites originated from 18 samples, all of which also carried the C16708G. To further evaluate the potential co-mutation pattern, we performed linear regression analysis and calculated Pearson correlation coefficients for iSNVs at these five positions. The results showed a strong correlation (*r* > 0.7) with high statistical significance (*P* < 2.5e-87; Fig. 2c and d). These findings indicated that these iSNVs contributed to a potential co-mutation pattern.

### Shared iSNVs across samples

**Fig 3:**
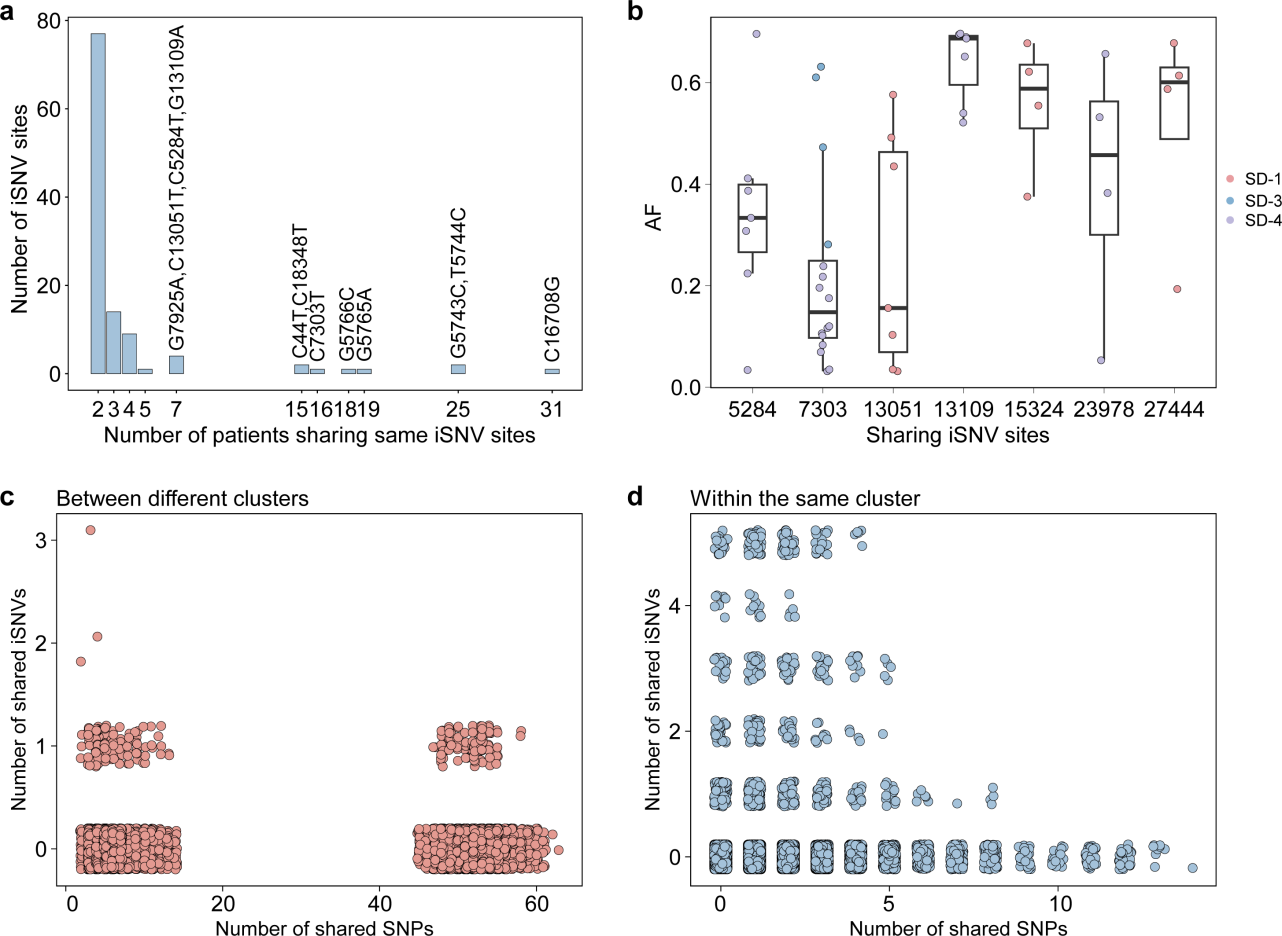
Shared iSNVs across samples. (**a**) Statistics of shared iSNVs. Each bar represents the number of iSNVs occurring in a given number of patients (≥2). iSNVs shared in >5 samples are shown on top of the bars. (**b**) Distribution of shared iSNVs with high AF in multiple samples. (**c**) Pairs of samples collected between different clusters. (**d**) Pairs of samples collected within the same cluster. Each unique pair is shown as a single point. The number of shared iSNVs by each pair is shown on the *y*-axis, with the number of SNP differences between the pair of genomes on the *x*-axis.

We investigated patterns of shared intra-host diversity between individuals. Most (1,041, 70.82%) iSNVs appeared in only a single sample. Among all iSNVs, 429 (29.18%) were present in at least two samples (Fig. 3a). The 197 iSNVs that occurred in more than five samples were not located at common sequencing error sites or homoplasious sites (32, 33). The shared iSNVs observed at seven positions had high AF across multiple samples, with only G13109A being a nonsynonymous iSNV (Fig. 3b). The G13109A was located in the ORF1ab gene and caused the amino acid aspartic acid (D) to be replaced by asparagine (N; D4282N). Additionally, among these seven mutations, the C7303T was present across different transmission clusters, indicating that shared iSNVs may arise independently in samples without epidemiological links.

To further explore the occurrence of shared iSNVs in sample pairs lacking epidemiological connections, we analyzed all unique sample pairs with detected iSNVs (*n* = 93,961). The majority of iSNVs were unique to samples, resulting in most sample pairs not sharing any iSNVs. Only 1.06% of the pairs (994 pairs) shared at least one iSNV. Of these pairs, 243 involved samples that belonged to different transmission clusters (Fig. 3c and d). Within these 243 pairs, 15 pairs showed SNP differences of just two to three and were collected within a ≤2-day interval. Additionally, 74% of these pairs were sequenced in different batches, lowering the likelihood that shared iSNVs were due to cross-contamination.

Co-infection cases can also confound transmission analysis based on shared iSNVs. An examination of AF at polymorphic sites in each sample confirmed that no co-infections were present in this study. This is likely because each transmission cluster was predominantly driven by a single sublineage strain.

### The evolution patterns from iSNVs to SNPs

**Fig 4:**
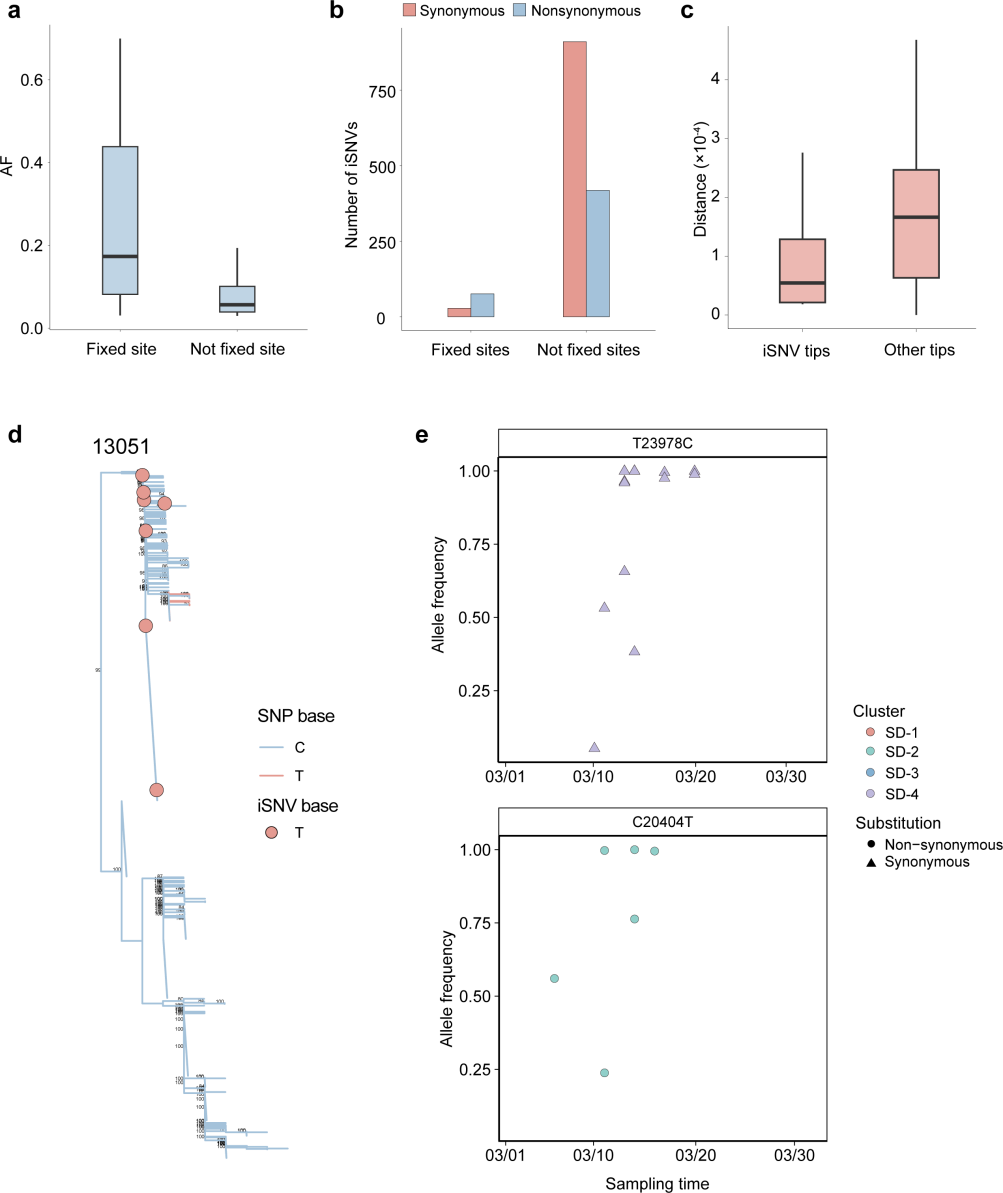
The evolution patterns from iSNVs to SNPs. (**a**) Distribution of AF for fixed and unfixed iSNVs. (**b**) Number of nonsynonymous and synonymous for fixed and unfixed iSNVs. (**c**) Box plot of patristic distances from iSNV tips and other tips to the nearest consensus branch change for 20 shared iSNV sites in the coding region. (**d**) The phylogeny where a consensus change is in close proximity to iSNVs with the relevant pair of nucleotides involved for the site 13,051. Tree branches are colored by the SNP at that position, and filled circles indicate iSNVs present in samples. For clarity, only support values > 80% are shown. (**e**) AF of mutation across samples. The mutations have been observed both as low-frequency variants and as fixed mutations.

To avoid misclassifying Omicron-featured mutations (BA.1.1, BA.2, and BA.2.3) as SNPs, we excluded all sites containing featured mutations prior to subsequent analysis. Among 1,253 genomic sites harboring mutations with AF ≥ 0.03, we identified 37 sites where an iSNV in one sample corresponded to an identical base pair substitution reaching SNP status in at least one other distinct sample. We define these sites as potential fixed sites. The fixed sites displayed a higher AF compared to non-fixed sites (Fig. 4a). The proportion of nonsynonymous iSNVs in fixed sites was lower than that of synonymous iSNVs (Fig. 4b). Meanwhile, the nonsynonymous iSNVs in fixed sites were only distributed in ORF1ab, S, and ORF3a genes.

We further investigated whether iSNVs can be used to resolve phylogenies and transmission clusters. If an iSNV evolves into an SNP through transmission, we would expect a phylogenetic association where samples containing the iSNV cluster with branches showing an SNP at the same sites. Of 113 sites shared across ≥2 samples, only 20 were fixed sites and located within protein-coding regions. For these 20 fixed iSNV sites, we observed that the patristic distance between a given iSNV position in one sample and the nearest sample with the same SNP was significantly lower than that of samples without the iSNV (*P* < 0.001; Fig. 4c). This suggested that iSNVs cluster within the phylogenetic tree, with branches supporting the same variants as SNPs.

When analyzing each site individually, 11 showed a significant association after applying the Benjamini-Hochberg correction (*P* < 0.05). For example, in site 13,051, the blue branches represent the SNP change from a C to T, with nearby iSNVs appearing as minor transitions at the ancestral nodes of the changing branches (Fig. 4d). Among 11 significant iSNV sites, we observed a gradual AF increase in C23978T and C20404T within a single transmission cluster (Fig. 4e). These findings suggested that iSNVs may act as a mutation reservoir for SNPs. We found that samples from the BA.1.1 sublineage carried iSNVs (C21618T and C26060T) at the defining positions of the BA.2 and BA.2.3 sublineages.

## DISCUSSION

The emergence of the divergent Omicron lineage pandemic is of worldwide concern. Characterizing the intra-host diversity of SARS-CoV-2 is crucial for identifying potential variants and inferring transmission dynamics (5, 34, 35). Our analysis characterized the genetic diversity of iSNVs and provided insights into the likelihood of shared iSNVs independently emerging in epidemiologically unlinked individuals, as well as the transition of iSNVs into SNPs.

Since the emergence of SARS-CoV-2, there have been numerous mutant strains, with a few exhibiting significantly enhanced transmissibility and infectivity (36–38). Studying the mutation patterns in the SARS-CoV-2 genome helps identify specific regions or genes that have undergone positive selection. Our results showed that the distribution of iSNVs was relatively uniform across most regions of the whole genome, while uneven in some regions, consistent with previous studies (25). Among all the genes, the N gene appears to be the most conserved gene during this outbreak, making it a potential target for vaccine development (39, 40). Specifically, we found that two regions, located in nsp3 and nsp13, accumulated more iSNVs and were subject to more selection pressure than other regions in the genome. They have a higher iSNV density compared to other regions primarily due to the presence of shared iSNVs, which form a potential co-mutation pattern, including G5743C, T5744C, G5765A, G5766C, and C16708G. The first four mutations were located in the papain-like protease (PLpro) encoded by nsp3, which cleaves the viral polyprotein for assembly and exhibits deubiquitination activity to evade the host’s innate immune response (41, 42). The C16708G was located on nsp13, which inhibits interferon production *in vivo* (43, 44). The accumulation of iSNVs on PLpro and nsp13 may affect the pathogenicity and immune escape ability of SARS-CoV-2.

Evaluating within-host viral diversity is crucial for understanding the transmission process. A narrow transmission bottleneck allows only a limited number of variants to successfully transmit between hosts. Our results revealed limited within-host diversity of SARS-CoV-2, suggesting insufficient selective pressure for the virus to adapt to the host environment. This limited diversity, together with our finding that most samples lack shared iSNVs, supports the existence of a narrow transmission bottleneck (10, 23). Such a bottleneck not only influences viral evolution but also restricts the utility of iSNVs for high-resolution transmission inference.

Shared iSNVs observed across genomes separated by time and distinct evolutionary lineages indicate their convergent emergence, complicating their use in transmission inference (17). In contrast to previous studies (35, 45), which faced challenges in establishing epidemiological connections, our samples, derived from four transmission clusters, allow us to better understand the extent of the emergence of convergent iSNVs. Our findings revealed that 24.4% of sample pairs sharing at least one iSNV originated from different transmission clusters. Moreover, even in cases where consensus sequences were highly similar and sampling dates were within 2 days apart, iSNV sharing can still occur between samples with no epidemiological links. Given that convergent evolution can significantly contribute to the emergence of shared iSNVs, caution should be exercised when using these shared iSNVs to infer transmission (46).

While convergent evolution may limit direct transmission inference from shared iSNVs, our findings demonstrate that, in certain cases, these iSNVs can still be transmitted successfully and significantly influence the evolution of phylogenetic consensus sequences (22, 23). Additionally, the frequencies of shared iSNV can be observed to gradually increase over time, becoming fixed in subsequent infections. These observations underscore the critical role of shared iSNVs in elucidating transmission dynamics. The iSNVs serve as a reservoir of mutations for potential epidemic variants (5, 47). Our results indicate that iSNVs detected in samples from earlier sublineages correspond to characteristic mutations observed in later-emerging sublineages, highlighting their important role in the formation of SNPs. This process may drive the evolution of new viral sublineages, especially those with enhanced transmissibility or virulence. Under specific high selective pressures, iSNVs can undergo adaptive evolution, transitioning from iSNVs to SNPs, which facilitates the virus’s ability to adapt and spread (48).

Our study has several limitations. First, although we detected iSNVs in early sublineage samples that are characteristic mutations of later sublineages, we did not observe the progression of these iSNVs becoming fixed SNPs. Therefore, future studies should increase the sample size and sampling duration to further investigate how variants of concern mutations and lineages become fixed as SNPs. Second, the biological functions of the identified potential co-mutations were not validated. Further studies are needed to determine whether these co-occurring iSNVs are adaptive mutations and how they affect viral function.

### Conclusions

In summary, our findings revealed that the significant enrichment of shared iSNVs in high-density regions drives the formation of potential co-mutation patterns. Although shared iSNVs are important, they alone are insufficient to reliably reconstruct transmission pathways due to the interference of convergent evolution. The iSNVs serve as mutation reservoirs, with iSNVs from early sublineages potentially developing into characteristic mutations in later sublineages. These insights enhanced our understanding of the mutational processes, evolutionary dynamics, and transmission mechanisms of SARS-CoV-2.

## Data Availability

The SARS-CoV-2 Omicron sequences were derived from a prior publication (18) and can be obtained from the corresponding author upon reasonable request.

## SUPPLEMENTAL MATERIAL

Supplemental figures msphere.00355-25-s0001.pdf Fig. S1 to S3.

ASM does not own the copyrights to Supplemental Material that may be linked to, or accessed through, an article. The authors have granted ASM a non-exclusive, world-wide license to publish the Supplemental Material files. Please contact the corresponding author directly for reuse.

## References

[1] Carabelli AM, Peacock TP, Thorne LG, Harvey WT, Hughes J, Peacock SJ, Barclay WS, de Silva TI, Towers GJ, Robertson DL, COVID-19 Genomics UK Consortium. 2023. SARS-CoV-2 variant biology: immune escape, transmission and fitness. Nat Rev Microbiol 21:162–177. doi:10.1038/s41579-022-00841-7 36653446 PMC9847462

[2] Wang L, Møhlenberg M, Wang P, Zhou H. 2023. Immune evasion of neutralizing antibodies by SARS-CoV-2 Omicron. Cytokine Growth Factor Rev 70:13–25. doi:10.1016/j.cytogfr.2023.03.001 36948931 PMC9985919

[3] Wolf JM, Wolf LM, Bello GL, Maccari JG, Nasi LA. 2023. Molecular evolution of SARS-CoV-2 from December 2019 to August 2022. J Med Virol 95:e28366. doi:10.1002/jmv.28366 36458547 PMC9877913

[4] Yamasoba D, Kimura I, Nasser H, Morioka Y, Nao N, Ito J, Uriu K, Tsuda M, Zahradnik J, Shirakawa K, et al.. 2022. Virological characteristics of the SARS-CoV-2 Omicron BA.2 spike. Cell 185:2103–2115. doi:10.1016/j.cell.2022.04.035 35568035 PMC9057982

[5] Sun F, Wang X, Tan S, Dan Y, Lu Y, Zhang J, Xu J, Tan Z, Xiang X, Zhou Y, He W, Wan X, Zhang W, Chen Y, Tan W, Deng G. 2021. SARS-CoV-2 quasispecies provides an advantage mutation pool for the epidemic variants. Microbiol Spectr 9:e0026121. doi:10.1128/spectrum.00261-21 34346744 PMC8552775

[6] Hou YJ, Chiba S, Halfmann P, Ehre C, Kuroda M, Dinnon KH, Leist SR, Schäfer A, Nakajima N, Takahashi K, et al.. 2020. SARS-CoV-2 D614G variant exhibits efficient replication ex vivo and transmission in vivo. Science 370:1464–1468. doi:10.1126/science.abe8499 33184236 PMC7775736

[7] Yurkovetskiy L, Wang X, Pascal KE, Tomkins-Tinch C, Nyalile TP, Wang Y, Baum A, Diehl WE, Dauphin A, Carbone C, Veinotte K, Egri SB, Schaffner SF, Lemieux JE, Munro JB, Rafique A, Barve A, Sabeti PC, Kyratsous CA, Dudkina NV, Shen K, Luban J. 2020. Structural and functional analysis of the D614G SARS-CoV-2 spike protein variant. Cell 183:739–751. doi:10.1016/j.cell.2020.09.032 32991842 PMC7492024

[8] Su YCF, Zeller MA, Cronin P, Zhang R, Zhuang Y, Ma J, Wong FY, Ng GGK, O’Toole Á, Rambaut A, Low JG, Smith GJD. 2025. Rapid emergence and evolution of SARS-CoV-2 intrahost variants among COVID-19 patients with prolonged infections, Singapore. Emerg Infect Dis 31:1537–1549. doi:10.3201/eid3108.241419 40592354 PMC12309778

[9] Pavia G, Quirino A, Marascio N, Veneziano C, Longhini F, Bruni A, Garofalo E, Pantanella M, Manno M, Gigliotti S, et al.. 2024. Persistence of SARS-CoV-2 infection and viral intra- and inter-host evolution in COVID-19 hospitalized patients. J Med Virol 96:e29708. doi:10.1002/jmv.29708 38804179

[10] Hannon WW, Roychoudhury P, Xie H, Shrestha L, Addetia A, Jerome KR, Greninger AL, Bloom JD. 2022. Narrow transmission bottlenecks and limited within-host viral diversity during a SARS-CoV-2 outbreak on a fishing boat. Virus Evol 8:veac052. doi:10.1093/ve/veac052 35799885 PMC9257191

[11] da Silva Filipe A, Shepherd JG, Williams T, Hughes J, Aranday-Cortes E, Asamaphan P, Ashraf S, Balcazar C, Brunker K, Campbell A, et al.. 2021. Genomic epidemiology reveals multiple introductions of SARS-CoV-2 from mainland Europe into Scotland. Nat Microbiol 6:112–122. doi:10.1038/s41564-020-00838-z 33349681

[12] Lu J, du Plessis L, Liu Z, Hill V, Kang M, Lin H, Sun J, François S, Kraemer MUG, Faria NR, et al.. 2020. Genomic epidemiology of SARS-CoV-2 in Guangdong Province, China. Cell 181:997–1003. doi:10.1016/j.cell.2020.04.023 32359424 PMC7192124

[13] Lane CR, Sherry NL, Porter AF, Duchene S, Horan K, Andersson P, Wilmot M, Turner A, Dougall S, Johnson SA, et al.. 2021. Genomics-informed responses in the elimination of COVID-19 in Victoria, Australia: an observational, genomic epidemiological study. Lancet Public Health 6:e547–e556. doi:10.1016/S2468-2667(21)00133-X 34252365 PMC8270762

[14] Song S, Li C, Kang L, Tian D, Badar N, Ma W, Zhao S, Jiang X, Wang C, Sun Y, et al.. 2021. Genomic epidemiology of SARS-CoV-2 in Pakistan. Genomics Proteomics Bioinformatics 19:727–740. doi:10.1016/j.gpb.2021.08.007 34695600 PMC8546014

[15] San JE, Ngcapu S, Kanzi AM, Tegally H, Fonseca V, Giandhari J, Wilkinson E, Nelson CW, Smidt W, Kiran AM, Chimukangara B, Pillay S, Singh L, Fish M, Gazy I, Martin DP, Khanyile K, Lessells R, de Oliveira T. 2021. Transmission dynamics of SARS-CoV-2 within-host diversity in two major hospital outbreaks in South Africa. Virus Evol 7:veab041. doi:10.1093/ve/veab041 34035952 PMC8135343

[16] Popa A, Genger J-W, Nicholson MD, Penz T, Schmid D, Aberle SW, Agerer B, Lercher A, Endler L, Colaço H, et al.. 2020. Genomic epidemiology of superspreading events in Austria reveals mutational dynamics and transmission properties of SARS-CoV-2. Sci Transl Med 12:eabe2555. doi:10.1126/scitranslmed.abe2555 33229462 PMC7857414

[17] Braun KM, Moreno GK, Wagner C, Accola MA, Rehrauer WM, Baker DA, Koelle K, O’Connor DH, Bedford T, Friedrich TC, Moncla LH. 2021. Acute SARS-CoV-2 infections harbor limited within-host diversity and transmit via tight transmission bottlenecks. PLoS Pathog 17:e1009849. doi:10.1371/journal.ppat.1009849 34424945 PMC8412271

[18] Xu Y, Liu T, Li Y, Wei X, Wang Z, Fang M, Zhang Y, Zhang H, Zhang L, Zhang J, Xu J, Tian Y, He N, Zhang Y, Wang Y, Yao M, Pang B, Wang S, Wen H, Kou Z. 2023. Transmission of SARS-CoV-2 Omicron variant under a dynamic clearance strategy in Shandong, China. Microbiol Spectr 11. doi:10.1128/spectrum.04632-22 PMC10101114 36916974

[19] Li H, Durbin R. 2010. Fast and accurate long-read alignment with Burrows-Wheeler transform. Bioinformatics 26:589–595. doi:10.1093/bioinformatics/btp698 20080505 PMC2828108

[20] Wang Y, Wang D, Zhang L, Sun W, Zhang Z, Chen W, Zhu A, Huang Y, Xiao F, Yao J, et al.. 2021. Intra-host variation and evolutionary dynamics of SARS-CoV-2 populations in COVID-19 patients. Genome Med 13:30. doi:10.1186/s13073-021-00847-5 33618765 PMC7898256

[21] Du P, Ding N, Li J, Zhang F, Wang Q, Chen Z, Song C, Han K, Xie W, Liu J, et al.. 2020. Genomic surveillance of COVID-19 cases in Beijing. Nat Commun 11:5503. doi:10.1038/s41467-020-19345-0 33127911 PMC7603498

[22] Li B, Deng A, Li K, Hu Y, Li Z, Shi Y, Xiong Q, Liu Z, Guo Q, Zou L, et al.. 2022. Viral infection and transmission in a large, well-traced outbreak caused by the SARS-CoV-2 Delta variant. Nat Commun 13:460. doi:10.1038/s41467-022-28089-y 35075154 PMC8786931

[23] Lythgoe KA, Hall M, Ferretti L, de Cesare M, MacIntyre-Cockett G, Trebes A, Andersson M, Otecko N, Wise EL, Moore N, et al.. 2021. SARS-CoV-2 within-host diversity and transmission. Science 372. doi:10.1126/science.abg0821 PMC8128293 33688063

[24] Schirmer M, D’Amore R, Ijaz UZ, Hall N, Quince C. 2016. Illumina error profiles: resolving fine-scale variation in metagenomic sequencing data. BMC Bioinformatics 17:125. doi:10.1186/s12859-016-0976-y 26968756 PMC4787001

[25] Li J, Du P, Yang L, Zhang J, Song C, Chen D, Song Y, Ding N, Hua M, Han K, et al.. 2022. Two-step fitness selection for intra-host variations in SARS-CoV-2. Cell Rep 38:110205. doi:10.1016/j.celrep.2021.110205 34982968 PMC8674508

[26] Al Khatib HA, Benslimane FM, Elbashir IE, Coyle PV, Al Maslamani MA, Al-Khal A, Al Thani AA, Yassine HM. 2020. Within-host diversity of SARS-CoV-2 in COVID-19 patients with variable disease severities. Front Cell Infect Microbiol 10:575613. doi:10.3389/fcimb.2020.575613 33123498 PMC7572854

[27] Zhou H-Y, Cheng Y-X, Xu L, Li J-Y, Tao C-Y, Ji C-Y, Han N, Yang R, Wu H, Li Y, Wu A. 2022. Genomic evidence for divergent co-infections of co-circulating SARS-CoV-2 lineages. Comput Struct Biotechnol J 20:4015–4024. doi:10.1016/j.csbj.2022.07.042 35915661 PMC9330581

[28] Otasek D, Morris JH, Bouças J, Pico AR, Demchak B. 2019. Cytoscape automation: empowering workflow-based network analysis. Genome Biol 20:185. doi:10.1186/s13059-019-1758-4 31477170 PMC6717989

[29] Katoh K, Standley DM. 2013. MAFFT multiple sequence alignment software version 7: improvements in performance and usability. Mol Biol Evol 30:772–780. doi:10.1093/molbev/mst010 23329690 PMC3603318

[30] Nguyen L-T, Schmidt HA, von Haeseler A, Minh BQ. 2015. IQ-TREE: a fast and effective stochastic algorithm for estimating maximum-likelihood phylogenies. Mol Biol Evol 32:268–274. doi:10.1093/molbev/msu300 25371430 PMC4271533

[31] Didelot X, Wilson DJ. 2015. ClonalFrameML: efficient inference of recombination in whole bacterial genomes. PLoS Comput Biol 11:e1004041. doi:10.1371/journal.pcbi.1004041 25675341 PMC4326465

[32] van Dorp L, Acman M, Richard D, Shaw LP, Ford CE, Ormond L, Owen CJ, Pang J, Tan CCS, Boshier FAT, Ortiz AT, Balloux F. 2020. Emergence of genomic diversity and recurrent mutations in SARS-CoV-2. Infect Genet Evol 83:104351. doi:10.1016/j.meegid.2020.104351 32387564 PMC7199730

[33] Issues with SARS-CoV-2 sequencing data. 2020. Virological. Available from: https://virological.org/t/issues-with-sars-cov-2-sequencing-data/473

[34] Lin G-L, Drysdale SB, Snape MD, O’Connor D, Brown A, MacIntyre-Cockett G, Mellado-Gomez E, de Cesare M, Bonsall D, Ansari MA, Öner D, Aerssens J, Butler C, Bont L, Openshaw P, Martinón-Torres F, Nair H, Bowden R, RESCEU Investigators, Golubchik T, Pollard AJ. 2021. Distinct patterns of within-host virus populations between two subgroups of human respiratory syncytial virus. Nat Commun 12:5125. doi:10.1038/s41467-021-25265-4 34446722 PMC8390747

[35] Pathak AK, Mishra GP, Uppili B, Walia S, Fatihi S, Abbas T, Banu S, Ghosh A, Kanampalliwar A, Jha A, et al.. 2022. Spatio-temporal dynamics of intra-host variability in SARS-CoV-2 genomes. Nucleic Acids Res 50:1551–1561. doi:10.1093/nar/gkab1297 35048970 PMC8860616

[36] Cheng L, Song S, Zhou B, Ge X, Yu J, Zhang M, Ju B, Zhang Z. 2021. Impact of the N501Y substitution of SARS-CoV-2 Spike on neutralizing monoclonal antibodies targeting diverse epitopes. Virol J 18:87. doi:10.1186/s12985-021-01554-8 33910569 PMC8081001

[37] Wang M, Zhang L, Li Q, Wang B, Liang Z, Sun Y, Nie J, Wu J, Su X, Qu X, Li Y, Wang Y, Huang W. 2022. Reduced sensitivity of the SARS-CoV-2 Lambda variant to monoclonal antibodies and neutralizing antibodies induced by infection and vaccination. Emerg Microbes Infect 11:18–29. doi:10.1080/22221751.2021.2008775 34818119 PMC8725979

[38] Li Q, Nie J, Wu J, Zhang L, Ding R, Wang H, Zhang Y, Li T, Liu S, Zhang M, et al.. 2021. SARS-CoV-2 501Y.V2 variants lack higher infectivity but do have immune escape. Cell 184:2362–2371. doi:10.1016/j.cell.2021.02.042 33735608 PMC7901273

[39] Abbasi H, Tabaraei A, Hosseini SM, Khosravi A, Nikoo HR. 2022. Real-time PCR Ct value in SARS-CoV-2 detection: RdRp or N gene? Infection 50:537–540. doi:10.1007/s15010-021-01674-x 34331262 PMC8323962

[40] Oronsky B, Larson C, Caroen S, Hedjran F, Sanchez A, Prokopenko E, Reid T. 2022. Nucleocapsid as a next-generation COVID-19 vaccine candidate. Int J Infect Dis 122:529–530. doi:10.1016/j.ijid.2022.06.046 35788417 PMC9250828

[41] Klemm T, Ebert G, Calleja DJ, Allison CC, Richardson LW, Bernardini JP, Lu BG, Kuchel NW, Grohmann C, Shibata Y, et al.. 2020. Mechanism and inhibition of the papain-like protease, PLpro, of SARS-CoV-2. EMBO J 39:e106275. doi:10.15252/embj.2020106275 32845033 PMC7461020

[42] Ferreira JC, Villanueva AJ, Al Adem K, Fadl S, Alzyoud L, Ghattas MA, Rabeh WM. 2024. Identification of novel allosteric sites of SARS-CoV-2 papain-like protease (PLpro) for the development of COVID-19 antivirals. J Biol Chem 300:107821. doi:10.1016/j.jbc.2024.107821 39342997 PMC11538808

[43] Feng K, Zhang H-J, Min Y-Q, Zhou M, Deng F, Wang H-L, Li P-Q, Ning Y-J. 2023. SARS-CoV-2 NSP13 interacts with host IRF3, blocking antiviral immune responses. J Med Virol 95:e28881. doi:10.1002/jmv.28881 37314155

[44] Yuen C-K, Lam J-Y, Wong W-M, Mak L-F, Wang X, Chu H, Cai J-P, Jin D-Y, To KK-W, Chan JF-W, Yuen K-Y, Kok K-H. 2020. SARS-CoV-2 nsp13, nsp14, nsp15 and orf6 function as potent interferon antagonists. Emerg Microbes Infect 9:1418–1428. doi:10.1080/22221751.2020.1780953 32529952 PMC7473193

[45] Tonkin-Hill G, Martincorena I, Amato R, Lawson ARJ, Gerstung M, Johnston I, Jackson DK, Park N, Lensing SV, Quail MA, et al.. 2021. Patterns of within-host genetic diversity in SARS-CoV-2. Elife 10:e66857. doi:10.7554/eLife.66857 34387545 PMC8363274

[46] Valesano AL, Rumfelt KE, Dimcheff DE, Blair CN, Fitzsimmons WJ, Petrie JG, Martin ET, Lauring AS. 2021. Temporal dynamics of SARS-CoV-2 mutation accumulation within and across infected hosts. PLoS Pathog 17:e1009499. doi:10.1371/journal.ppat.1009499 33826681 PMC8055005

[47] Lemieux JE, Siddle KJ, Shaw BM, Loreth C, Schaffner SF, Gladden-Young A, Adams G, Fink T, Tomkins-Tinch CH, Krasilnikova LA, et al.. 2021. Phylogenetic analysis of SARS-CoV-2 in Boston highlights the impact of superspreading events. Science 371:eabe3261. doi:10.1126/science.abe3261 33303686 PMC7857412

[48] Nijhuis M, van Maarseveen NM, Boucher CAB. 2009. Antiviral resistance and impact on viral replication capacity: evolution of viruses under antiviral pressure occurs in three phases. Handb Exp Pharmacol, no. 189:299–320. doi:10.1007/978-3-540-79086-0_11 19048205

