# Supplementary material for: Intra-host variation and transmission dynamics of SARS-CoV-2 Omicron outbreaks in Shandong, China
Source: mSphere. 2025 Sep 22;10(10):e00355-25. doi: 10.1128/msphere.00355-25 (PMC12570505; doi:10.1128/msphere.00355-25)

Supplementary Figures

Supplementary Figure 1 Box pots showing distribution of allele frequencies (AF).

Supplementary Figure 2 Quality control for iSNV detection.

Supplementary Figure 3 Network analysis of samples with shared iSNV sites.

Supplementary Figure 1. Box pots showing distribution of allele frequencies (AF). The x-axis gives the nucleotide position, with the number of samples AF ≥ 3% in brackets.

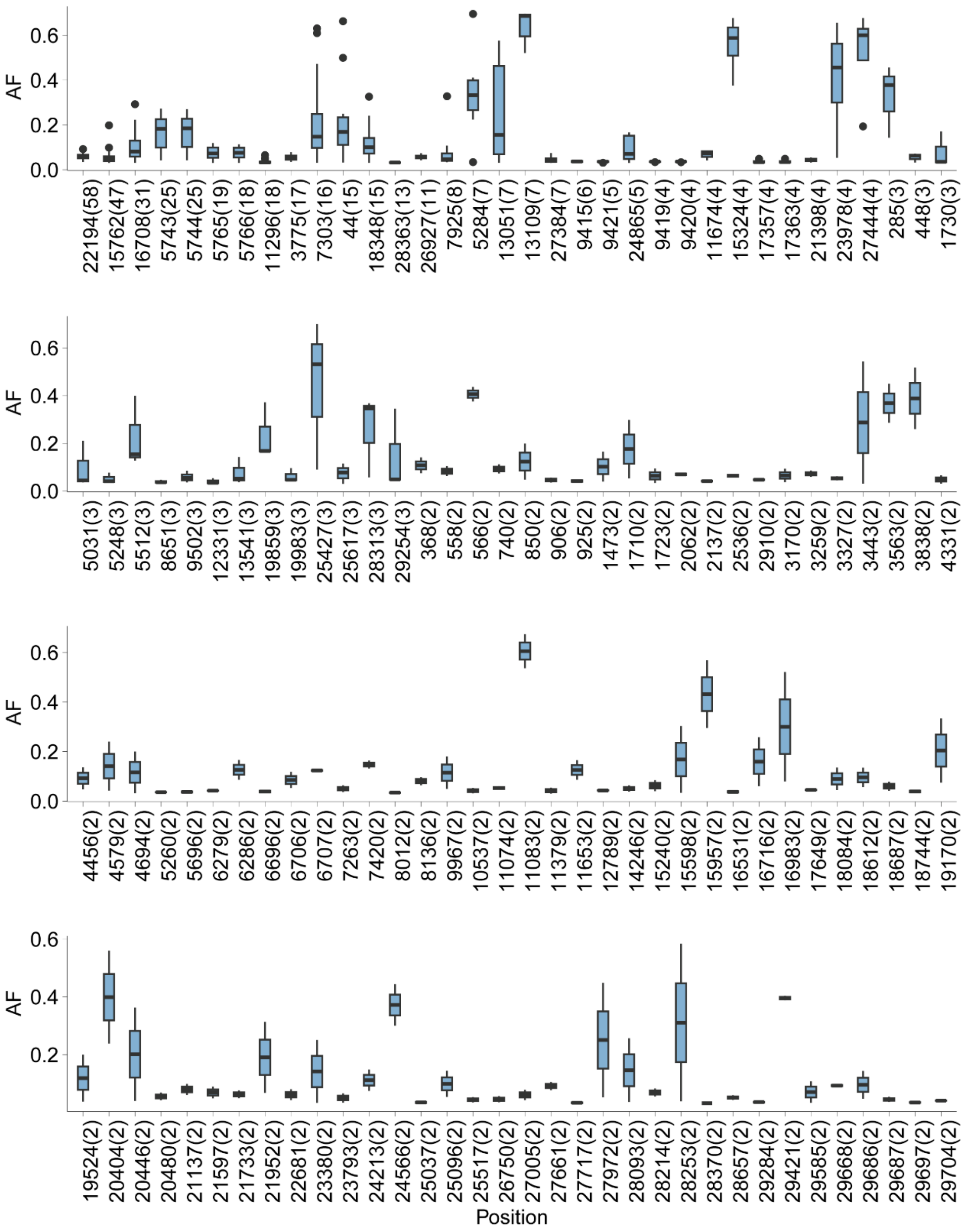

Supplementary Figure 2. Quality control for iSNV detection. a) Correlation between the number of iSNVs and the the mean sequencing depth (log10) for sequenced samples. b) Correlation between the number of iSNVs and cycle threshold (Ct) values (ORF1ab) for sequenced samples. c) Correlation between the number of iSNVs and Ct values (N) for sequenced samples.

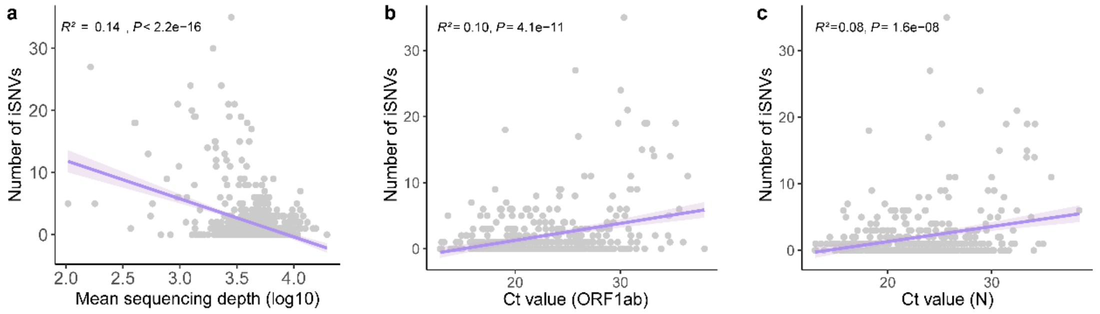

Supplementary Figure 3. Network analysis of patients with shared iSNV sites. Nodes denote samples and edges indicate that two samples have the same iSNV site.

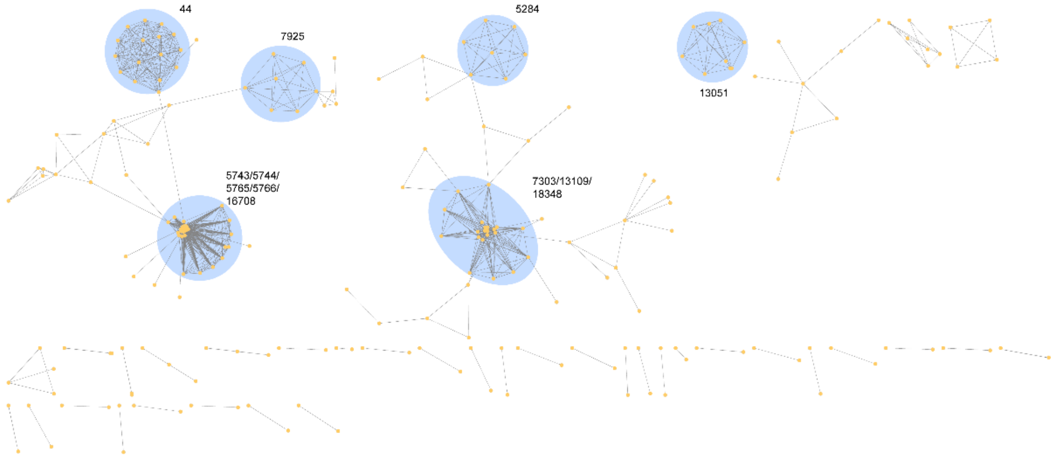

Supplement: Supplemental figures — Fig. S1 to S3. [file msphere.00355-25-s0001.pdf]
